# Social support and care burden in family caregivers of patients with hematologic malignancies: a parallel mediation model of coping strategies

**DOI:** 10.7717/peerj.21527

**Published:** 2026-07-21

**Authors:** Xiaohe Fan, Yu Zhang, Jingsong Yan, Zhijie Kang, Yue Wei, Xiaotong Guo, Yikun Liu, Junfeng Chen, Ding Ding

**Affiliations:** 1 School of Public Health, Dalian Medical University, Dalian, Liaoning, China; 2 Department of Hematology, The Second Hospital of Dalian Medical University, Dalian, Liaoning, China

**Keywords:** Hematologic malignancies, Care burden, Social support, Family caregivers

## Abstract

**Objective:**

With the rising incidence of hematologic malignancies (HMs) in recent years, caregivers of HMs patients have increasingly emerged as a significant factor affecting the treatment of these conditions. This study aimed to explore the association of coping strategies on the relationship between care burden and social support among family caregivers of HMs patients.

**Methodology:**

A questionnaire survey was conducted involving 207 family caregivers of hospitalized HMs patients in a tertiary hospital in Dalian from October 2023 to May 2024 and 200 valid responses were collected. We used hierarchical linear regression and parallel mediation model to assess the relationships and potential mediating effects among care burden, coping strategies, and social support.

**Results:**

In our study, the care burden showed a medium level and average score was 44.21 ± 15.92. Positive coping strategies (*r* =  − 0.769, *P* < 0.01) and social support (*r* =  − 0.654, *P* < 0.01) showed a negative correlation with care burden, while negative coping strategies was significantly correlated with care burden (*r* = 0.788, *P* < 0.01). According to the mediating effect analysis after covariate adjustment, the relationships between social support and care burden were mediated by coping strategies in total samples and the indirect effect value was −0.955, accounted for 73.57% of the total effect.

**Conclusions:**

Family caregivers of HMs patients with more negative coping strategies and less social support were significantly associated with higher levels of care burden. Coping strategies served as a partial parallel mediator between social support and care burden.

## Introduction

Hematologic malignancies (HMs) encompass disorders of the hematopoietic system, including lymphoma, leukemia, and myelodysplastic syndromes. It is estimated that about 178,000 new cases of lymphoma, leukemia, and multiple myeloma are diagnosed globally every year (Entezari et al., 2024). In China, taking several common HMs as examples, approximately 80 thousand new cases of leukemia and 30 thousand new cases of multiple myeloma were reported in 2022 (Han et al., 2022). HMs are characterized by complex disease courses and a high risk of complications, which often require sustained involvement from family caregivers and may consequently increase care burden (Nielsen et al., 2022). Care burden refers to the negative physical, psychological, social, and economic impacts experienced by caregivers while providing care for patients (Zhang et al., 2014). When such burden accumulates, it may adversely affect caregivers’ health and well-being and further compromise the quality of care provided to patients (Yang et al., 2023). Previous studies have shown that about 63% of caregivers have depressive tendencies, and that care burden, unemployment, and financial concerns are important factors associated with depression (Wang et al., 2016), which is emphasized between family caregivers and their patients. A survey conducted by the American Association of Retired Persons (AARP) revealed that family caregivers constituted more than 80% of all caregivers worldwide (National Alliance for Caregiving, 2020). This pattern is also occurring in the treatment of HMs, where family caregivers often play a central role in daily care, treatment accompaniment, and emotional support (Schulz et al., 2020; Caprini & Motta, 2021). Therefore, care burden among family caregivers of patients with HMs deserves close attention.

This burden has important implications for caregivers’ well-being. In China, 70% of family caregivers reported experiencing care burden and stress in 2021. Previous studies have shown that excessive care burden may cause physiological and psychological problems among caregivers, including weight changes, cognitive impairment, increased anxiety, and reduced well-being (Zhang et al., 2023). In the Chinese context, caregiving is deeply shaped by traditional family-centered values, particularly filial piety and a strong sense of family responsibility. Such values may strengthen caregivers’ sense of moral obligation and emotional commitment to care. However, when internalized too rigidly, they may also discourage disclosure of distress and help-seeking, increasing the risk of sustained burden. This cultural context may help explain why social support is not only directly related to care burden, but may also influence it through caregivers’ coping responses (Zhang, Sun & Yan, 2024). Persistent burden may also undermine caregivers’ ability to provide effective care, thereby affecting patients’ recovery and quality of life (Adelman et al., 2014). In this context, resources that can buffer caregivers’ ongoing strain become especially important. Among these, social support has been widely recognized as a key protective factor related to care burden (Gallicchio et al., 2002; Vincent et al., 2009).

Social support is defined as the informational, emotional, and instrumental assistance individuals receive from their social networks. It can be categorized into formal and informal support (Lu et al., 2020). Charlton, McQuaid & Wallace (2023) reported that social support can alleviate stress and protect caregivers from the adverse effects of stressful life events; nevertheless, approximately 60% of caregivers still report at least five moderate or high unmet needs. Another study indicated that only 17% of family caregivers reported receiving strong social support (Nemcikova, Katreniakova & Nagyova, 2023). Family caregivers with inadequate social support often lack opportunities to relieve caregiving stress, which may leave their emotional needs unmet and impair their quality of life (Zhang et al., 2021). Previous studies have also shown that social support is an important contributor to quality of life (Morrison et al., 2020). During the caregiving process, support and connections from others may help alleviate care burden and improve psychological outcomes and quality of life among caregivers (Nielsen et al., 2022).

Although social support is widely associated with caregiver well-being, care burden remains substantial among family caregivers of patients with HMs. In China, where family members often sustain care across treatment and recovery, caregivers’ support resources and coping capacity may influence not only their own burden but also the continuity of cancer care (Sheng et al., 2024). This broader significance is underscored by China’s current cancer-control agenda under Healthy China 2030, a national health strategy that aims to raise the overall 5-year cancer survival rate to 46.6% by 2030 and promotes people-centered, whole-process care (Qi et al., 2023). Beyond screening and standardized treatment, current reforms emphasize multidisciplinary and disease-specific service models, rehabilitation, pain management, long-term care, nutritional and psychological support, stronger medical security, and improved access to anti-tumor drugs. In this context, caregiver burden is not merely an individual psychosocial outcome, but also a practical constraint on the delivery of high quality family-based cancer care. Examining whether social support alleviates care burden through coping strategies may therefore clarify how family caregivers can be better supported within China’s evolving cancer care system.

However, important gaps remain in understanding how social support influences care burden among family caregivers of patients with HMs. Existing studies have largely focused on general cancer populations, and few have examined whether coping strategies mediate the relationship between social support and care burden in this specific caregiving group (Karacan et al., 2021). Addressing this gap may help clarify the mechanism linking social support to caregiver outcomes and inform more supportive and human-centered cancer care for affected families in China. Therefore, this study aimed to examine the association between social support and care burden and to test the mediating role of coping strategies among family caregivers of patients with HMs in China.

## Materials & Methods

### Research design

A cross-sectional study was conducted to investigate the mediating role of coping strategies in the association between social support and care burden among family caregivers of patients with HMs. Data were collected using a questionnaire survey. The questionnaire included demographic information and scales assessing care burden, social support, and coping strategies. The demographic characteristics of family caregivers and patients included various factors such as gender, age, marital status, educational attainment, and specific factors like ethnicity, employment status, per capita monthly income, daily caregiving duration, and overall family dynamics for caregivers. For patients, relevant demographic factors included disease type, medical insurance type, and history of bone marrow transplant. Data were collected between October 2023 and May 2024. This design enabled the simultaneous assessment of social support, coping strategies, and care burden within the same group of caregivers, providing an appropriate framework for testing the proposed parallel mediation model.

### Participants

Participants were recruited from a tertiary teaching hospital in Dalian, Liaoning Province, China. The hospital is a major center for hematologic disease treatment and bone marrow transplantation in the region, and its Department of Hematology serves as the only provincial-level bone marrow transplantation center in Liaoning Province. In 2023, the department had 235 inpatient beds. Given its large volume of patients with HMs and its specialized hematology services, this hospital was considered an appropriate site for participant recruitment. A convenience sampling method was used to select primary family caregivers of hospitalized patients with HMs from the tertiary hospital between October 2023 and May 2024. The inclusion criteria for caregivers in this study were as follows: (1) Patients diagnosed with HMs through pathology (such as acute and chronic leukemia, lymphoma, multiple myeloma, *etc.*); (2) Caregivers who assumed primary caregiving responsibilities during the patients’ hospitalization (defined as providing the longest average daily caregiving time for at least seven consecutive days, excluding professional nursing staff). (3) Caregivers aged 18 or older and had a familial relationship with the patient. (4) Caregivers had normal communication abilities and no cognitive impairments; (5) The caregiver provided informed consent to participate in the study. Exclusion criteria were:(1) Caregivers of patients diagnosed with conditions not requiring long-term care; (2) Caregivers (such as nurses) have an employment relationship with the patients; (3) Caregivers with cognitive, mental, or communication problems; (4) Caregivers unwilling to participate in the survey. A total of 207 questionnaires were distributed. Excluding seven incomplete responses, 200 valid responses were finally collected, resulting in an effective response rate of 96.62%.

## Measure

### Care burden

The Care Burden Inventory (CBI), developed by Novak and Guest in 1989, includes five dimensions: physical, social, emotional, time dependence, and developmental impact, totaling 24 items. This scale employs a five-point scoring system, where each item is rated from 0 to 4 points, resulting in a total score range of 0 to 96. Higher scores indicate a greater burden on the caregiver. A sample item from the developmental burden dimension is: “I feel that I am missing out on life.” The CBI has been translated and adapted into Chinese, and the Chinese version has demonstrated good reliability and validity, with a Cronbach’s α coefficient of 0.92 and a test-retest reliability of 0.93 (Liu, Chen & Hu, 2023). As a multidimensional assessment tool, it has been widely used among caregivers across various disease populations in China.

### Social support

This study adopted the Social Support Rate Scale (SSRS) (Xiao, 1994). This scale includes three dimensions: subjective support, objective support, and support utilization, with a total of 10 items. The total score ranges from 12 to 66 points, with higher scores indicating higher levels of social support. Subjective support reflects the perceived availability of an individual’s social network. Objective support indicates the level of assistance received in emergencies in the past. Support utilization refers to the behavioral patterns individuals employ when seeking social support. A sample item is: “How many close friends do you have who can provide you with support and help?” The scale has a Cronbach’s α coefficient ranging from 0.825 to 0.896, indicating good reliability.

### Coping strategies

We used the Simplified Coping strategies Questionnaire (SCQ), developed by Xie (1998), to evaluate the level of coping strategies. The SCQ was derived from Ways of Coping Questionnaire (WCQ), developed by Folkman & Lazarus (1988). After its introduction to China, the WCQ was revised and simplified by Xie (1998) and generated the SCQ. This scale consists of two dimensions, positive coping and negative coping, and uses a four-point Likert scale with 20 items in total. Sample items include: “Try to see the bright side of things” (positive coping, Item 1) and “Smoke or drink to relieve distress”. The first dimension consists of items 1 to 12, which focus on positive coping tendencies; the second dimension consists of items 13 to 20, which emphasize negative coping tendencies. A higher average score supports a stronger inclination towards the respective coping strategies. The overall Cronbach’s α coefficient for the scale is 0.90.

### Procedures

Data were collected through a face-to-face questionnaire survey. Trained research staff approached potential participants in the hospital wards, explained the purpose and procedures of the study, and assured them of the confidentiality of their responses. After obtaining written informed consent, the research staff provided the questionnaires and were available to clarify any questions. On average, it took participants approximately 15–20 min to complete the questionnaire. Patients or the public were not involved in the design, conduct, reporting, or dissemination plans of our research. The study protocol was approved by the Human Ethics Committee of Dalian Medical University. The Ethics approval number was 2023004.

### Statistical analysis

Data analysis was performed using SPSS 28.0 (IBM Corp., Armonk, NY, USA) and AMOS 24.0. Firstly, descriptive statistics (frequency, percentage, mean, standard deviation, *etc.*) were used to measure caregivers’ demographic characteristics, care burden, social support, and coping strategies scores. T-tests and chi-square tests were utilized to evaluate the demographic differences between groups. Secondly, Pearson correlation analysis was conducted to explore the relationships among the three variables: social support, care burden and coping strategies. Subsequently, hierarchical regression analysis was used to test mediating effects of coping strategies. Finally, the bootstrap method was used to compute bias-corrected 95% confidence intervals based on 5,000 bootstrap samples, thereby assessing the mediating effects of coping strategies on the relationship between social support and care burden. The *P*-value is two-tailed, with statistical significance set at *P* < 0.05.

Because *a priori* power analysis was not conducted before data collection, a *post-hoc* sensitivity analysis was performed using G*Power 3.1 to assess the adequacy of the current sample size. Based on a sample size of 200, an alpha level of 0.05, and a desired power of 0.80, the analysis indicated that the study was able to detect a small effect size in the regression model. This suggests that the sample size was acceptable for testing the primary associations and mediation model in the present study.

## Results

### Demographic characteristics and difference in care burden of caregivers and patients

Among the 200 participants, the average care burden was 44.21 ± 15.92. The gender distribution revealed that the number of female caregivers was twice that of male caregivers. The caregivers’ average age was 50.89 ± 10.91 years. 50% of caregivers had an education level of junior high school or above. The majority of caregivers were married (accounting for 93%). Additionally, 91% of caregivers were lineal relation of patients, and half of caregivers reported a monthly household income ranging from 3,000 to 5,000 RMB. Most were not operating (65%), and a majority of caregivers (57%) provided care for more than 12 h each day. About half of caregivers cared only one patient. More caregivers had no health problem and appeared average in self-rate status. Analysis of the relationship between caregivers’ characteristics and care burden showed that care burden was significantly affected by caregivers’ household per capita monthly income, caregiver identity, self-rated health status, and changes in caregivers’ health status (Table 1).

**Table 1:** Demographic characteristics and univariate analysis of caregivers’ factors affecting care burden.

Variable		*N*	Mean ± SD	F/t	*P*
**Gender**				0.657	0.512
Female		130	44.75 ± 15.80		
Male		70	43.20 ± 16.22		
**Ethnicity**				0.425	0.671
Han		190	44.10 ± 16.15		
Others		10	46.30 ± 11.08		
**Age**				1.156	0.332
≤30		5	42.80 ± 9.63		
31–40		30	44.30 ± 18.39		
41–50		65	46.88 ± 16.17		
51–60		54	40.50 ± 16.47		
61–70		42	45.57 ± 13.60		
>70		4	37.75 ± 6.65		
**Marital status**				0.551	0.578
Single		7	44.00 ± 9.17		
Married		186	43.98 ± 16.27		
Divorced or Widowed		7	50.43 ± 10.89		
**Education level**				1.019	0.363
Junior High School and Below		96	45.85 ± 16.03		
High School/Vocational School		47	43.15 ± 15.24		
Associate Degree/Bachelor’s Degree and Above		57	42.32 ± 16.28		
**Per capita monthly income**				10.248	<0.001
<3,000 Yuan		51	54.02 ± 12.74		
3,000–5,000 Yuan		99	41.51 ± 14.75		
5,000–7,000 Yuan		43	40.21 ± 17.77		
>7,000 Yuan		7	35.57 ± 12.41		
**Daily care hours**				3.567	0.030
<8 hours		43	38.63 ± 17.42		
8–12 hours		44	44.77 ± 12.55		
>12 hours		113	46.12 ± 16.14		
**Have someone else to take care**				1.578	0.116
Yes		96	42.51 ± 14.85		
No		104	46.05 ± 16.90		
**Working situation**				2.962	0.054
Incumbency		71	40.58 ± 15.97		
Resignation or otherwise		129	46.21 ± 15.60		
**Identity**				3.547	0.016
Spouse		87	44.23 ± 15.62		
Children		54	41.09 ± 17.02		
Parents		41	50.49 ± 14.75		
Other Individuals		18	39.17 ± 12.91		
**Self-rated health status**				15.154	<0.001
Good		73	37.16 ± 14.28		
Average		121	47.63 ± 15.37		
Poor		6	61.00 ± 11.85		

The patients in this study had a mean age of 53.42 ± 18.70 years. The number of male and female patients was approximately equal. About 75% of patients were married. Half of patients have educational level in junior high school or below. Leukemia was the top in disease diagnosis, and most of patients had not been transplanted in the past. As shown in Table 2, care burden differed significantly according to patient age, marital status, and disease diagnosis (*P* < 0.05).

**Table 2:** Demographic characteristics and univariate analysis of patients’ factors affecting care burden.

Variable			N	Mean ± SD	F/t	*P*
**Gender**				0.384	0.702
Male			109	44.61 ± 16.02		
Female			91	43.74 ± 15.88		
**Marital status**					6.349	0.002
Single			36	50.44 ± 14.39		
Married			149	43.75 ± 15.72		
Divorced or Widowed			15	33.80 ± 15.97		
**Age**				2.600	0.010
≤10			2	49.00 ± 19.80		
11-20			16	52.38 ± 14.86		
21-30			11	48.09 ± 16.54		
31-40			24	50.54 ± 17.12		
41-50			21	43.19 ± 16.03		
51-60			40	42.10 ± 16.01		
61-70			53	43.83 ± 13.16		
71-80			27	40.70 ± 17.47		
>80			6	25.17 ± 9.66		
**Education level**					0.810	0.446
Junior High School and Below			106	42.07 ± 16.17		
High School/Vocational School			37	44.83 ± 15.06		
Junior College/Undergraduate or Above			57	42.51 ± 18.03		
**Disease Diagnosis**					15.442	<0.001
Leukemia			116	50.49 ± 14.40		
Lymphoma			25	42.00 ± 12.00		
Myelodysplastic Syndromes			23	38.83 ± 13.26		
Multiple Myeloma			15	30.40 ± 10.49		
Aplastic Anemia			11	32.82 ± 15.72		
Others			10	22.50 ± 10.08		
**Type of medical insurance**					0.480	0.632
Medical insurance for urban workers			91	44.80 ± 15.33		
Medical insurance for urban residents			109	43.72 ± 16.46		
**Whether Transplanted**					1.354	0.177
Yes			43	47.12 ± 14.88		
No			157	43.41 ± 16.15		

### Pearson correlation analysis of study variable

The results of the correlation coefficients for the variables in this study were shown in Table 3. Social support was positively correlated with positive coping (*r* = 0.766, *P* < 0.01) and negatively correlated with negative coping (*r* =  − 0.654, *P* < 0.01) and care burden (*r* =  − 0.724, *P* < 0.01). Furthermore, care burden was negatively correlated with positive coping (*r* =  − 0.769, *P* < 0.01) and positively correlated with negative coping (*r* = 0.788, *P* < 0.01).

**Table 3:** Correlation analysis of care burden.

Variable	Social support	Positive coping	Negative coping	Care burden
Social support	1			
Positive coping	0.766^**^	1		
Negative coping	−0.654^**^	−0.722^**^	1	
Care burden	−0.724^**^	−0.769^**^	0.788^**^	1

**Notes.**

** *P* < 0.01 (two-tailed).

### Hierarchical linear regression of care burden

The regression results for social support, coping strategies, and care burden were presented in Table 4. Step 1 measured the impact of demographic variables and self-rated variables related to care burden identified in univariate tests. Step 2 showed the direct effect of social support on care burden. After controlling demographic variables, social support was found to influence care burden (*t* =  − 13.270, *P* < 0.01). When the mediating variables of positive and negative coping were included, Step 3 demonstrated the parallel mediating effect of coping strategies (including positive and negative coping) when the independent variable was social support. Positive coping (*t* =  − 2.907, *P* < 0.05), negative coping (*t* = 8.750, *P* < 0.01), and social support all affected care burden (*t* =  − 1.796, *P* < 0.05). The partial regression coefficient associated with social support increased from −0.612 to −0.112, suggesting that coping strategies may serve as a partial mediator between social support and care burden. *R^2^* change from step1 to step2 was statistically significant (*P* < 0.01). After coping strategies were added in Step 3, the *R^2^* increased further compared with Step 2. In model 3, the VIF values for all variables are shown in Table 5, and all are below 10, indicating relatively low multicollinearity among the variables.

**Table 4:** Hierarchical linear regression analysis of factors associated with care burden.

	Care Burden					
	Step1		Step2		Step3	
Variable	*β*	t	*β*	t	*β*	t
Patient age	−0.270	−2.849^*^	−0.171	−2.508^*^	−0.063	−1.179
Marital status						
Unmarried (reference)						
Married	−0.025	−0.219	−0.056	−0.693	−0.127	−2.020
Divorced or Widowed	−0.051	−0.566	−0.016	−0.242	−0.061	−1.209
Disease diagnosis						
Leukemia (reference)						
Lymphoma	−0.109	−1.864	−0.059	−1.389	−0.046	−1.378
Myelodysplastic Syndromes	−0.196	−3.454^*^	−0.115	−2.810^*^	−0.116	−3.582^*^
Multiple Myeloma	−0.310	−5.511^*^	−0.233	−5.727^*^	−0.205	−6.383^*^
Aplastic Anemia	−0.289	−5.019^*^	−0.164	−3.863^*^	−0.187	−5.670^*^
Others	−0.295	−5.212^*^	−0.186	−4.482^*^	−0.197	−6.138^*^
Caregiver’s Family Monthly Income per Capita	−0.201	−3.236^*^	−0.053	−1.168	−0.066	−1.847
Caregiver’s Identity						
Other Personnel (reference)						
Caregiver’s Spouse	0.093	0.880	0.082	1.084	0.118	1.983^*^
Caregiver’s Child	0.223	2.282^*^	0.063	0.891	0.114	2.051^*^
Caregiver’s Parent	0.013	0.109	0.006	0.065	0.049	0.723
Caregiver’s Self-Rated Health Status						
Self-Rated Health Good (reference)						
Self-Rated Health Average	0.215	3.548^*^	0.063	1.412	0.026	0.731
Self-Rated Health Poor	0.183	3.154^*^	0.082	1.942	0.039	1.172
Social Support			−0.612	−13.270^*^	−0.112	−1.796
Positive Coping					−0.185	−2.907^*^
Negative Coping					0.466	8.750^*^
R^2^	0.460		0.724		0.836	
ΔR^2^	0.460		0.264		0.112	
F	11.243		32.165		54.508	

**Notes.**

* *P* < 0.05.

**Table 5:** Multicollinearity diagnostics for predictor variables in the final regression model (model 3) of factors associated with care burden.

**Predictor variable**	**Maximum VIF value**
Patient age	4.158
Patient marital status	3.274
Disease diagnosis	1.334
Caregiver identity	4.158
Self-rated health	1.629
Caregiver’s family monthly income per capita	2.607
Social support	4.543
Positive coping	4.715
Negative coping	3.474

**Notes.**

VIF Variance Inflation Factor

All values are below the conventional threshold of 10, indicating no severe multicollinearity in the final regression model (Model 3).

### Path analysis for structural model

**Figure 1:**
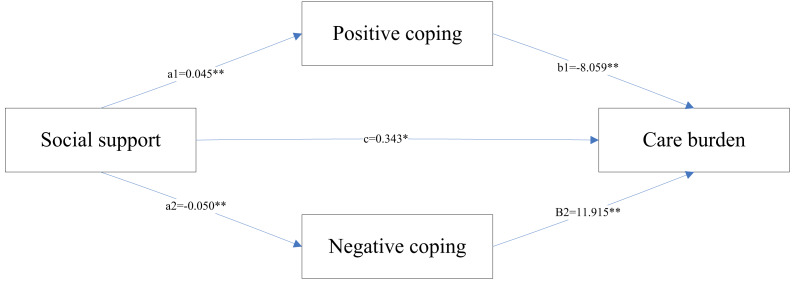
Parallel mediation model of the association between social support and care burden through coping strategies. Note: *P < 0.05, **P < 0.01 (two-tailed).

Finally, we used the bootstrap method with percentile bias correction to test the mediating effect of coping strategies between social support and care burden (Fig. 1 and Table 6). The results demonstrated that the total indirect effect of coping strategies was −0.955 (Bootstrap 95% CI [−1.252 to −0.697]), accounting for 73.57% of the total effect (total effect = −1.298). This mediating effect consisted of two specific pathways: the indirect effect through positive coping was −0.365 (Bootstrap 95% CI [−0.628 to −0.118]), and the indirect effect through negative coping was −0.590 (Bootstrap 95% CI [−0.766 to −0.437]). The detailed results are presented in Table 6.

These results illustrated that coping strategies had a partial mediating effect between social support and care burden, which had both direct effects and mediating effects. Social support had a direct impact on care burden and also influenced it through the mediating variable of coping strategies.

## Discussion

In this study, we explored the mediating effects of coping strategies in the relationship between social support and care burden among family caregivers. The average score of care burden was 44.21 ± 15.92, indicating a moderate level of burden. In the demographic characteristics, most caregivers (76.5%) reported a moderate or higher level of care burden, suggesting that the overall burden in this group was considerable. These findings align with the views of Chadda, Singh & Ganguly (2007), who emphasized the substantial burden often endured by family caregivers. Additionally, the fact that over half of the caregivers spent more than 12 h per day providing care also implies a heavy caregiving burden. In the Chinese hospital context, family caregivers often remain closely involved in patients’ daily care and treatment accompaniment, which may partly explain the long caregiving hours and elevated burden observed in this study. 64.5% of caregivers did not have stable employment, which may reflect the difficulty of balancing work with prolonged caregiving responsibilities. Furthermore, a pattern was broadly consistent with previous studies showing that caregivers often report greater use of positive than negative coping strategies (Yang et al., 2022). This suggests that participants tended to maintain a more positive attitude when confronting stress, possibly because caregivers believed that positive coping strategies could positively influence patients’ attitudes toward their illnesses.

Hierarchical linear regression was used to further examine factors associated with care burden after the univariate analyses. Among all the variables, disease diagnosis remained significant across all regression steps, even after social support and coping strategies were included in the model. The result was consistent with the study by Keykhaei et al. (2021) and may reflect differences in treatment intensity, prognosis, and long-term care demands across disease categories. For example, leukemia often involves prolonged treatment, intensive monitoring, and substantial financial costs, all of which may increase caregivers’ psychological strain, time demands, and economic pressure. As social support and coping strategies were added, predictive power of patient age actually declined and finally lost statistical significance in the final model. This finding was in line with the study by Xu et al. (2024) which suggested that age may not independently predict care burden when caregivers do not differ substantially in role-related conflict. Before coping strategies were added to the model, social support showed the largest effect size among the variables, highlighting its importance in alleviating care burden. However, after including coping strategies, the effect size of social support decreased substantially, while coping strategies emerged as the most influential factor affecting care burden.

In our study, as anticipated, social support manifested a significant negative correlation (*r* =  − 0.724, *P* < 0.01) with the care burden among caregivers of patients with HMs, consistent with previous findings by Ashghali Farahani et al. (2021) and Nemcikova, Katreniakova & Nagyova (2023). Emotional, instrumental, and informational social support were the most frequently cited forms of support for caregivers (Wong et al., 2014). According to the theory of the stress-buffering effects of social support and the main effects theory (Ditzen & Heinrichs, 2014), this phenomenon may be attributed to the fact that social support can reduce the negative impact of stress on health through physiological mechanisms. Social support was associated with reward-relevant and anxiety-reducing structures and transmitter systems, which can decrease stress reactivity and anxiety. Inadequate social support can lead to a decline in quality of life and reduce caregivers’ capacity to manage care burden (Eisenberger et al., 2007). However, because of the cross-sectional design, the direction of this association cannot be determined. It is equally plausible that caregivers with higher levels of burden may be less likely to perceive available support, and may also be less inclined to adopt positive coping strategies. Thus, the relationship among social support, coping strategies, and caregiver burden may be bidirectional. Nevertheless, the findings highlight the importance of providing accessible professional guidance and supportive resources for family caregivers.

Additionally, our study found a significant correlation between the coping strategies and care burden. This finding was similar to those reported by Huang et al. (2015). Negative coping strategies, such as avoidance and resignation, could lower individuals’ physical and mental functioning, increasing the probability of depression and anxiety (Shakeri et al., 2015). Excessive care burden often resulted in physical and mental exhaustion for caregivers, which diminished their psychological resources and energy for taking positive coping strategies. When confronted with long-term and irreversible health conditions, like HMs, caregivers may experience feelings of helplessness and hopelessness. This emotional state could drive them to adopt negative coping strategies as a means of alleviating their internal pain and stress. In addition, according to the model proposed by Li and Chang, adopting positive coping strategies not only enhanced caregivers’ problem-solving abilities, but also improved self-confidence and happiness (Li et al., 2021). These factors contribute to a reduction in care burden (Hanly et al., 2016). On the other hand, this association may also be interpreted through the Chinese cultural lens of family obligation. Family obligation may motivate caregivers to adopt positive coping strategies, as caring for a family member aligns with deeply held values (Hu, Hu & Xu, 2023). However, when the obligation is internalized as an inflexible obligation, caregivers may feel compelled to conceal their distress and avoid seeking help, thereby reinforcing negative coping patterns. This cultural paradox suggests that the mediating role of coping strategies operates within a broader normative context that can either buffer or amplify care burden. Therefore, developing a positive coping strategies is helpful for caregivers to alleviate stress and improve the family’s collective resilience in facing challenges.

**Table 6:** Total, direct, and indirect effects of social support on care burden.

**Effects**	**Paths**	**SE**	**Estimate**	**Bootstrap 95% CI**	***P***
**Total effect**	SS→ CB	0.078	−1.298	−1.446 to −1.139	<0.001
**Direct effect**	SS→ CB	0.136	−0.343	−0.661 to −0.006	0.048
**Indirect effect**	SS→ PC→ CB	0.130	−0.365	−0.628 to −0.118	0.002
	SS→ NC→ CB	0.084	−0.590	−0.766 to −0.437	<0.001
**Total indirect effect**	Total indirect effect	0.140	−0.955	−1.252 to −0.697	<0.001

**Notes.**

SS social support PC positive coping NC negative coping CB care burden CI confidence interval

More importantly, this study showed that coping strategies served as mediating variables in the relationship between social support and care burden among family caregivers of patients with HMs, with a total indirect effect of −0.955, accounting for 73.57% of the total effect. This finding indicated that social support not only directly affected care burden but also indirectly affected it through both positive and negative coping strategies, which is consistent with previous research (Shao et al., 2020). A study involved 173 participants revealed that without enough social support, caregivers were more likely to take negative coping strategies, like avoidance, when facing problems beyond their capability (Wan et al., 2023). Negative coping strategies may reduce caregivers’ pain tolerance and physical functioning, thereby increasing discomfort during caregiving and undermining psychological resilience and well-being. On one hand, when caregivers obtain enough social support, they have more resources to deal with different difficulties. They may perceive these difficulties as more understandable and manageable, which can strengthen self-confidence and encourage the use of more positive coping strategies. On the other hand, positive coping strategies under pressure can promote an optimistic attitude reducing the possibility of their anxiety and care burden. Moreover, the “model of thriving through relational support”, proposed by Feeney and Collins, suggested that social support could encourage individuals to adopt positive coping strategies when facing stressful events. These positive coping strategies play a critical role in enhancing psychological resilience and improving caregivers’ quality of life. In the context of Chinese family caregiving for hematologic malignancies, this pathway may be especially salient because caregivers often rely heavily on family-based resources when facing prolonged treatment demands.

## Practical Implications

The present findings have several implications for clinical practice and supportive care. Routine psychosocial assessment for family caregivers of patients with HMs should evaluate not only care burden, but also perceived social support and coping patterns. This may help healthcare providers identify caregivers at higher risk of distress at an earlier stage and provide timely support. In addition, hospital-based supportive care programs could incorporate brief coping-oriented interventions, caregiver education, peer-support groups, and referral pathways for psychosocial assistance in order to strengthen adaptive coping and reduce reliance on negative coping responses. Because caregiver stress may fluctuate during treatment, future intervention models may also benefit from more dynamic forms of support, such as mobile health tools that monitor caregiver stress and provide timely guidance when needed. In the Chinese context, these strategies may also support ongoing efforts under the Healthy China 2030 initiative to strengthen supportive care, promote more people-centered services, and improve the overall quality of cancer care.

## Limitations

This study has several limitations. First, the cross-sectional design precludes causal inferences regarding the relationships among social support, coping strategies, and care burden. Second, the sample was recruited through convenience sampling from a single tertiary care hospital, which may limit the representativeness of the sample and the generalizability of the findings to caregivers in other clinical or community settings. The absence of *a priori* power analysis and the limited sample size not only constrained the feasibility of robust subgroup analyses across different hematologic malignancy types but also raised concerns about potential overfitting in the regression models, given the number of predictors relative to the sample size. Third, all variables were assessed using self-report measures from the same respondents at a single time point, which may increase the risk of response bias and common method variance. Future longitudinal, multicenter studies with larger and more diverse samples are needed to confirm and extend these findings.

## Conclusion

This study examined the relationship of social support, coping strategies, and care burden among family caregivers of patients with HMs. A parallel mediating model revealed that coping strategies (including both positive and negative coping strategies) played a mediating role between social support and care burden. Family caregivers who had more social support and greater tendency to use positive coping strategies reported lower care burden. Future interventions should therefore be culturally attuned, supporting caregivers in ways that respect the meaningful aspects of family responsibility while also encouraging more adaptive coping and timely help-seeking to reduce care burden. From a broader perspective, integrating caregiver support and coping-oriented interventions into routine cancer care may also contribute to current health reform priorities under the Healthy China 2030 initiative, particularly the promotion of people-centered, whole-process, and higher-quality hematologic care.

## Supplemental Information

10.7717/peerj.21527/supp-1 Supplemental Information 1 The Raw Data File in English

10.7717/peerj.21527/supp-2 Supplemental Information 2 The variable dictionary of raw data

10.7717/peerj.21527/supp-3 Supplemental Information 3 The anonymized raw data of this study

## Additional Information and Declarations

### Data Availability

The following information was supplied regarding data availability: The data is available in the Supplemental Files. The data is available at Zenodo: Fan, X. (2025). Social Support and Care Burden in Family Caregivers of Patients with Hematologic Malignancies: A Parallel Mediation Model of Coping Strategies [Data set]. Zenodo. https://doi.org/10.5281/zenodo.17767757.
