# Supplementary material for: Social support and care burden in family caregivers of patients with hematologic malignancies: a parallel mediation model of coping strategies
Source: PeerJ. 2026 Jul 21;14:e21527. doi: 10.7717/peerj.21527 (PMC13398390; doi:10.7717/peerj.21527)
Supplement: Supplemental Information 2 [file peerj-14-21527-s002.pdf]

Variable Dictionary for the Revised Raw Data File

Study: Social Support and Care Burden in Family Caregivers of Patients with Hematologic Malignancies

This document explains the English variable names used in the revised raw-data workbook and the coding of each variable. The English wording was aligned as closely as possible with the questionnaire and the manuscript, and missing item labels were translated into concise academic English for data-sharing purposes.

Important note: The original hospitalization number column was removed from the revised raw-data workbook for de-identification. The deleted questionnaire item (original export header a13) was blank and is not included in the revised Raw_Data sheet. Checkbox/multiple-response variables were standardized to 1/0 (1 = selected/yes, 0 = not selected/no).

Alignment note with the manuscript

The wording in this variable dictionary was checked against the manuscript Methods section. Instrument names are reported here using the manuscript's terminology: Care Burden Inventory (CBI), Social Support Rate Scale (SSRS), and Simplified Coping strategies Questionnaire (SCQ).

How to read the social support variables

The manuscript describes the SSRS as a 10-item scale. In the raw-data file, item-level coding is retained. Multi-response source items were expanded into separate binary variables so that the raw-data file is transparent and machine-readable for reviewers.

1. Administrative variables

Variable name	Description	Coding / value labels
record_id	Study record ID	Sequential record number

2. Caregiver demographic variables

Variable name	Description	Coding / value labels
caregiver_birth_ym	Caregiver birth year and month	Text, format YYYY.MM
caregiver_gender	Caregiver gender	1=Male; 2=Female
caregiver_ethnicity	Caregiver ethnicity	1=Han; 2=Other
caregiver_marital_status	Caregiver marital status	1=Single; 2=Married; 3=Divorced; 4=Widowed
caregiver_occupation	Caregiver occupation	1=Government/public institution/enterprise manager; 2=Professional/technical personnel; 3=Clerical and related personnel; 4=Commercial/service personnel; 5=Agriculture/forestry/animal husbandry/fishery worker; 6=Production/manufacturing worker; 7=Military personnel; 8=Student; 9=Freelancer/self-employed; 10=Unemployed; 11=Other
caregiver_employment_status	Caregiver employment status	1=Employed; 2=Not employed (including retired/unemployed/laid off); 3=Other

caregiver_education_level	Caregiver education level	1=Junior high school and below; 2=High school/technical secondary school; 3=College/Bachelor; 4=Master and above
caregiver_monthly_income_pc	Per-capita monthly household income of caregiver family	1=<3000 RMB; 2=3000-5000 RMB; 3=5000-7000 RMB; 4=>7000 RMB

3. Patient diagnosis and treatment-related variables

Variable name	Description	Coding / value labels
patient_diagnosis	Patient diagnosis	1=Acute lymphoblastic leukemia; 2=Acute myeloid leukemia; 3=Chronic lymphocytic leukemia; 4=Chronic myeloid leukemia; 5=Myelodysplastic syndromes; 6=Aplastic anemia; 7=Multiple myeloma; 8=Lymphoma; 9=Myeloproliferative neoplasms; 10=Thrombocytopenia; 11=Other
patient_disease_duration_months	Months since patient diagnosis	Numeric (months; may include decimals such as 0.5)
payment_self_pay	Medical payment method: self-pay	1=Selected/Yes; 0=Not selected/No
payment_urban_employee_insurance	Medical payment method: urban employee medical insurance	1=Selected/Yes; 0=Not selected/No
payment_urban_resident_insurance	Medical payment method: urban/rural resident medical insurance	1=Selected/Yes; 0=Not selected/No
payment_commercial_insurance	Medical payment method: commercial medical insurance	1=Selected/Yes; 0=Not selected/No
prior_hsct_status	Previous hematopoietic stem cell transplantation (HSCT)	1=Allogeneic transplant; 2=Autologous transplant; 3=No transplant

4. Caregiving context variables

Variable name	Description	Coding / value labels
caregiver_relationship_to_patient	Relationship of caregiver to patient	1=Spouse; 2=Child; 3=Parent; 4=Sibling; 5=Other
caregiving_duration_months	Months of caregiving provided so far	Numeric (months; may include decimals such as 0.5)
daily_care_hours	Approximate daily caregiving time during hospitalization	1=<4 hours; 2=4-8 hours; 3=8-12 hours; 4=>12 hours
other_caregivers_count	Number of other people helping care for the patient	1=None; 2=One person; 3=Two or more persons
other_family_members_need_care	Other family members also needing care	1=No; 2=Yes

5. Caregiver health-related variables

Variable name	Description	Coding / value labels
caregiver_diagnosed_health_problem	Caregiver has a doctor-diagnosed health problem	1=No; 2=Yes
caregiver_self_rated_health	Caregiver self-rated current health status	1=Very good; 2=Good; 3=Average; 4=Poor; 5=Very poor
caregiver_health_change_after_caregiving	Change in caregiver health compared with before caregiving	1=No change; 2=Somewhat worse; 3=Much worse

6. Coping strategies variables (SCQ)

Variable name	Description	Coding / value labels
coping_01_work_study_activity	Solve problems through work, study, or other activities	0=Never; 1=Occasionally; 2=Often; 3=Always
coping_02_talk_with_others	Talk with others and share inner distress	0=Never; 1=Occasionally; 2=Often; 3=Always
coping_03_see_positive_side	Try to see the positive side of things	0=Never; 1=Occasionally; 2=Often; 3=Always
coping_04_change_thinking_priorities	Change the way I think and rediscover what matters in life	0=Never; 1=Occasionally; 2=Often; 3=Always
coping_05_not_take_too_seriously	Do not take the problem too seriously	0=Never; 1=Occasionally; 2=Often; 3=Always
coping_06_stick_to_own_stance	Stick to my own stance	0=Never; 1=Occasionally; 2=Often; 3=Always
coping_07_find_multiple_solutions	Find several different ways to solve the problem	0=Never; 1=Occasionally; 2=Often; 3=Always
coping_08_seek_advice	Seek advice from relatives, friends, or classmates	0=Never; 1=Occasionally; 2=Often; 3=Always
coping_09_change_own_practices	Change some of my original practices or my own problems	0=Never; 1=Occasionally; 2=Often; 3=Always
coping_10_learn_from_others	Learn from how others handled similar difficult situations	0=Never; 1=Occasionally; 2=Often; 3=Always
coping_11_hobbies_sports	Develop hobbies and actively participate in cultural/sports activities	0=Never; 1=Occasionally; 2=Often; 3=Always
coping_12_suppress_negative_emotions	Try to suppress disappointment, regret, sadness, and anger	0=Never; 1=Occasionally; 2=Often; 3=Always
coping_13_rest_or_vacation	Try to rest or take a vacation and temporarily put the problem aside	0=Never; 1=Occasionally; 2=Often; 3=Always
coping_14_smoke_drink_eat_medicate	Relieve distress by smoking, drinking, taking medication, or eating	0=Never; 1=Occasionally; 2=Often; 3=Always
coping_15_wait_for_time_to_change	Believe time will change the situation and just wait	0=Never; 1=Occasionally; 2=Often; 3=Always
coping_16_try_to_forget	Try to forget the whole thing	0=Never; 1=Occasionally; 2=Often; 3=Always
coping_17_rely_on_others	Rely on others to solve the problem	0=Never; 1=Occasionally; 2=Often; 3=Always
coping_18_accept_reality	Accept reality because there is no other way	0=Never; 1=Occasionally; 2=Often; 3=Always
coping_19_hope_for_miracle	Imagine that some miracle may change the current situation	0=Never; 1=Occasionally; 2=Often; 3=Always
coping_20_comfort_myself	Comfort myself	0=Never; 1=Occasionally; 2=Often; 3=Always

7. Care burden variables (CBI)

Variable name	Description	Coding / value labels
care_burden_01_help_daily_living	The patient needs my help with most activities of daily living	0=Never; 1=Occasionally; 2=Sometimes; 3=Often; 4=Always
care_burden_02_patient_dependence	The patient is very dependent on me	0=Never; 1=Occasionally; 2=Sometimes; 3=Often; 4=Always
care_burden_03_needs_company	The patient constantly needs my company	0=Never; 1=Occasionally; 2=Sometimes; 3=Often; 4=Always
care_burden_04_help_basic_activities	I have to help the patient complete many basic activities	0=Never; 1=Occasionally; 2=Sometimes; 3=Often; 4=Always
care_burden_05_hardly_rest	I can hardly rest while caring for the patient	0=Never; 1=Occasionally; 2=Sometimes; 3=Often; 4=Always
care_burden_06_cannot_enjoy_life	I cannot enjoy my own life	0=Never; 1=Occasionally; 2=Sometimes; 3=Often;

		4=Always
care_burden_07_want_change_life	I wish I could change my current life situation	0=Never; 1=Occasionally; 2=Sometimes; 3=Often; 4=Always
care_burden_08_social_life_affected	My social life has been affected	0=Never; 1=Occasionally; 2=Sometimes; 3=Often; 4=Always
care_burden_09_emotionally_exhausted	I feel emotionally exhausted because of caregiving	0=Never; 1=Occasionally; 2=Sometimes; 3=Often; 4=Always
care_burden_10_not_expected_life	This is not the life I had expected	0=Never; 1=Occasionally; 2=Sometimes; 3=Often; 4=Always
care_burden_11_insufficient_sleep	I do not get enough sleep	0=Never; 1=Occasionally; 2=Sometimes; 3=Often; 4=Always
care_burden_12_health_affected	My health has been affected	0=Never; 1=Occasionally; 2=Sometimes; 3=Often; 4=Always
care_burden_13_physically_uncomfortable	Caring for the patient makes me physically uncomfortable	0=Never; 1=Occasionally; 2=Sometimes; 3=Often; 4=Always
care_burden_14_physically_drained	I feel physically drained	0=Never; 1=Occasionally; 2=Sometimes; 3=Often; 4=Always
care_burden_15_family_less_harmonious	My relationship with family is not as harmonious as before	0=Never; 1=Occasionally; 2=Sometimes; 3=Often; 4=Always
care_burden_16_family_not_understand	My family does not fully understand my efforts in caring for the patient	0=Never; 1=Occasionally; 2=Sometimes; 3=Often; 4=Always
care_burden_17_work_performance_worse	My work performance is not as good as before	0=Never; 1=Occasionally; 2=Sometimes; 3=Often; 4=Always
care_burden_18_dislike_nonhelping_relatives	I dislike relatives who do not help	0=Never; 1=Occasionally; 2=Sometimes; 3=Often; 4=Always
care_burden_19_confused_by_patient_behavior	I feel confused by some of the patient's behaviors	0=Never; 1=Occasionally; 2=Sometimes; 3=Often; 4=Always
care_burden_20_family_embarrassment	Having such a patient in the family makes me feel embarrassed and hard to cope	0=Never; 1=Occasionally; 2=Sometimes; 3=Often; 4=Always
care_burden_21_dislike_caring	I do not like caring for my patient	0=Never; 1=Occasionally; 2=Sometimes; 3=Often; 4=Always
care_burden_22_uncomfortable_with_visitors	I feel uncomfortable when friends come to visit	0=Never; 1=Occasionally; 2=Sometimes; 3=Often; 4=Always
care_burden_23_angry_when_communicating	I often feel angry when communicating with the patient	0=Never; 1=Occasionally; 2=Sometimes; 3=Often; 4=Always
care_burden_24_marital_problems	My marriage has developed problems	0=Never; 1=Occasionally; 2=Sometimes; 3=Often; 4=Always

8. Social support variables (SSRS)

Variable name	Description	Coding / value labels
social_support_01_close_friends_count	Close friends who can provide support/help	1=None; 2=1-2; 3=3-5; 4=6 or more
social_support_02_family_relationship	Closeness of relationship with family in the past year	1=Far from family and living alone; 2=Residence often changes and mostly lives with strangers;

		3=Lives with classmates/colleagues/friends; 4=Lives with family
social_support_03_neighbor_relationship	Relationship with neighbors	1=No concern, only nodding acquaintance; 2=May show a little concern when in difficulty; 3=Some neighbors care about you; 4=Most neighbors care about you
social_support_04_colleague_friend_relationship	Relationship with colleagues/friends	1=No concern, only nodding acquaintance; 2=May show a little concern when in difficulty; 3=Some colleagues/friends care about you; 4=Most colleagues/friends care about you
social_support_05_support_spouse_partner	Support/care received from spouse/partner	1=None; 2=Very little; 3=Average; 4=Full support
social_support_06_support_parents	Support/care received from parents	1=None; 2=Very little; 3=Average; 4=Full support
social_support_07_support_children	Support/care received from children	1=None; 2=Very little; 3=Average; 4=Full support
social_support_08_support_siblings	Support/care received from siblings	1=None; 2=Very little; 3=Average; 4=Full support
social_support_09_support_other_family	Support/care received from other family members	1=None; 2=Very little; 3=Average; 4=Full support
social_support_10_any_economic_practical_support	Any source of economic support or practical help in emergencies (past)	1=None; 2=Has one or more sources
social_support_11_economic_support_spouse	Economic/practical support source: spouse	1=Selected/Yes; 0=Not selected/No
social_support_12_economic_support_other_family	Economic/practical support source: other family members	1=Selected/Yes; 0=Not selected/No
social_support_13_economic_support_relatives	Economic/practical support source: relatives	1=Selected/Yes; 0=Not selected/No
social_support_14_economic_support_friends	Economic/practical support source: friends	1=Selected/Yes; 0=Not selected/No
social_support_15_economic_support_colleagues	Economic/practical support source: colleagues	1=Selected/Yes; 0=Not selected/No
social_support_16_economic_support_work_unit	Economic/practical support source: work unit	1=Selected/Yes; 0=Not selected/No
social_support_17_economic_support_official_org	Economic/practical support source: official or semi-official organizations	1=Selected/Yes; 0=Not selected/No
social_support_18_economic_support_nonofficial_org	Economic/practical support source: religious/social/non-official organizations	1=Selected/Yes; 0=Not selected/No
social_support_19_economic_support_other	Economic/practical support source: other	1=Selected/Yes; 0=Not selected/No
social_support_20_any_emotional_support	Any source of comfort and care in emergencies (past)	1=None; 2=Has one or more sources
social_support_21_emotional_support_spouse	Comfort/care source: spouse	1=Selected/Yes; 0=Not selected/No
social_support_22_emotional_support_other_family	Comfort/care source: other family members	1=Selected/Yes; 0=Not selected/No
social_support_23_emotional_support_relatives	Comfort/care source: relatives	1=Selected/Yes; 0=Not selected/No
social_support_24_emotional_support_friends	Comfort/care source: friends	1=Selected/Yes; 0=Not selected/No
social_support_25_emotional_support_colleagues	Comfort/care source: colleagues	1=Selected/Yes; 0=Not selected/No
social_support_26_emotional_support_work_unit	Comfort/care source: work unit	1=Selected/Yes; 0=Not selected/No
social_support_27_emotional_support_official_org	Comfort/care source: official or semi-official organizations	1=Selected/Yes; 0=Not selected/No
social_support_28_emotional_support_nonofficial_org	Comfort/care source: religious/social/non-official organizations	1=Selected/Yes; 0=Not selected/No
social_support_29_emotional_support_other	Comfort/care source: other	1=Selected/Yes; 0=Not selected/No
social_support_30_distress_disclosure_willingness	Willingness to talk about troubles	1=Never tell anyone; 2=Only tell 1-2 very close people; 3=Would tell if friends ask; 4=Actively tell others to obtain support/understanding
social_support_31_help_seeking_frequency	Frequency of seeking help when troubled	1=Only rely on myself; 2=Rarely ask others for help;

		3=Sometimes ask others for help; 4=Often seek help from family/friends/organizations when in difficulty
social_support_32_group_activity_participation	Participation in group activities	1=Never participate; 2=Occasionally; 3=Often; 4=Actively participate and play an active role

9. Patient demographic variables

Variable name	Description	Coding / value labels
patient_birth_ym	Patient birth year and month	Text, format YYYY.MM
patient_gender	Patient gender	1=Male; 2=Female
patient_marital_status	Patient marital status	1=Single; 2=Married; 3=Divorced; 4=Widowed
patient_education_level	Patient education level	1=Primary school and below; 2=Junior high school; 3=High school/technical secondary school; 4=College diploma; 5=Bachelor; 6=Master and above
patient_occupation	Patient occupation	1=Government/public institution/enterprise manager; 2=Professional/technical personnel; 3=Clerical and related personnel; 4=Commercial/service personnel; 5=Agriculture/forestry/animal husbandry/fishery worker; 6=Production/manufacturing worker; 7=Military personnel; 8=Student; 9=Freelancer/self-employed; 10=Unemployed; 11=Other

Appendix. Variables not included in the revised Raw_Data sheet

Original header	Original meaning	Reason excluded	Suggested note for rebuttal/data note
aa	Hospitalization number	Removed from output workbook for de-identification	Removed to protect participant privacy before public sharing.
a13	Deleted questionnaire item (Q13)	Blank in the export; not included in output workbook	Blank deleted item in the original questionnaire export.
